# Effect of Urchin-Like Mullite Whiskers on the High-Temperature Performance of Porous SiO_2_-Based Ceramic Molds

**DOI:** 10.3390/ma12071181

**Published:** 2019-04-11

**Authors:** Yi Chen, Zhongliang Lu, Weijian Wan, Jian Li, Kai Miao, Dichen Li

**Affiliations:** 1 State Key Laboratory of Manufacturing Systems Engineering, School of Mechanical Engineering, Xi’an Jiaotong University, Xi’an 710049, China; chenyi0706@foxmail.com (Y.C.); wanweijian1992@163.com (W.W.); lijianxjtu@yeah.net (J.L.); lidcxjtu@163.com (D.L.); 2 Collaborative Innovation Center for Advanced Aero-Engine, XueYuan Road No.37, HaiDian District, Beijing 100191, China

**Keywords:** urchin-like mullite whiskers, high-temperature strength, SiO_2_ ceramic, Al additive, gelcasting

## Abstract

Urchin-like mullite whiskers synthesized by the vapor–liquid–solid growth method were used to improve the high-temperature performance of porous gelcast SiO_2_-based ceramic molds. Aluminum was used to facilitate the synthesis of polycrystal urchin-like mullite whiskers which acted as bridges between particles. Scanning electron microscopy (SEM) and X-ray diffraction (XRD) were used to investigate the microstructures and phase compositions of the sintered ceramic samples, respectively. Urchin-like mullite whiskers with diameters of 0.2~1.0 µm and lengths of 1.0~8.0 µm were successfully synthesized in SiO_2_-based ceramic. When 15 vol% Al was added, the high-temperature strength at 1200 °C was improved from 8.5 to 27.5 MPa, and the creep deformation was decreased to 0.56 mm. Meanwhile, a sintering shrinkage below 0.3% was obtained, and the de-coring rate was accelerated by 67% compared to that of the pure SiO_2_-based ceramic. This method showed excellent high-temperature strength and high precision, having remarkable potential in the fabrication of hollow turbine blades.

## 1. Introduction

SiO_2_-based ceramic molds are widely used for casting hollow turbine blades for gas turbines and aircraft engines [1] because of their low density, good leachability, and excellent creep resistance [2,3,4]. Li put forward the integral fabrication technique (IFT) to manufacture ceramic molds with complex internal cores by stereolithography (SL) and gelcasting methods [5]. In particular, the SiO_2_ ceramic molds are exposed to liquid superalloy with a temperature over 1200 °C while casting. Premature cracking, total collapse, and mold warpage [6] usually occur due to the low high-temperature strength of ceramic molds, resulting in molten metal leakage or a less precise blade [7].

In fact, many methods, including mineralizer addition [8], high solid loading [4], infiltration strengthening [9], as well as fiber or whisker addition [10,11,12], have been used to improve the high-temperature strength of SiO_2_-based ceramic molds in much research. Mullite whisker reinforcement is one of the most effective high-temperature strengthening methods due to its numerous advantages, including high melting point, chemical inertness, and excellent comprehensive mechanical properties [13,14,15]. Mullite whiskers can be either added to or synthesized in SiO_2_-based ceramic. The limited amount of added mullite whiskers would affect the viscosity and reduce the filling ability of ceramic slurry. Therefore, much research has turned to synthesizing mullite whiskers through gel or powder calcination, mineral decomposition, and molten salt-assisted synthesis methods [16,17]. For example, Park [18] developed mullite whiskers by firing appropriate mixtures of coal fly ash and NH_4_Al(SO_4_)_2_. Li [19] discussed the effect of foaming agents on the development of mullite whiskers. Mao [20] introduced the vapor–liquid–solid (VLS) growth mechanism which revealed the synthesis of whiskers. Nevertheless, few reports about the high-temperature strength of gelcast SiO_2_-based ceramic molds reinforced by VLS synthesized mullite whiskers have appeared in the literature. Compared with the methods mentioned above, the VLS growth method is a low-cost and more effective way to strengthen SiO_2_ ceramic through whiskers synthesized in situ for the better dispersion of whiskers and it does not require external material additives [21].

Al_2_O_3_ plays an essential role in the formation of the mullite phase at high temperatures in the SiO_2_-based ceramic system. Mullite whiskers can be easily developed when a proper amount of SiO_2_ and Al_2_O_3_ is well mixed in the ceramic matrix [21,22,23]. Previous research [6] proved that Al has a high reaction activity. The oxidation of Al proceeds as both solid/gas and liquid/gas reactions which could provide the VLS growth conditions [24]. Hence, it can be considered a sintering additive to facilitate the formation of mullite in SiO_2_-based ceramic. Meanwhile, Al powder has a low melting point, and the existence of liquid and gas phases in high-temperature sintering can provide good kinetic and thermodynamic conditions to favor the growth of mullite whiskers in the processing of VLS growth [21]. The morphological controlling of mullite is possible by adjusting the Al content in the SiO_2_-based ceramic system [21,24].

In this study, SiO_2_ with different particle sizes were used to prepare ceramic molds by combining SL and gelcasting techniques. Al was adopted as a sintering assistant to synthesize urchin-like mullite whiskers using the VLS growth method. The growth mechanism of urchin-like mullite whiskers was proposed. The effects of urchin-like mullite whiskers on the high-temperature performance of SiO_2_-based ceramic molds were investigated, and the strengthening mechanism of high-temperature strength was also discussed.

## 2. Materials and Methods

### 2.1. Raw Materials

In this study, aspheric-fused SiO_2_ powders with different particle sizes (*D*_50_ = 100, 40, 5, 2 µm, Shandong Zibo Aluminum Ltd., Zibo, China) were used as the matrix material. Acrylamide (AM), N,N′-methylenebisacrylamide (MBAM), N,N,N′,N′-tetramethylethylenediamine (C_6_H_16_N_2_), ammonium persulfate ((NH4)_2_S_2_O_8_), and sodium polyacrylate (Xi’an Tianyi Chemical Reagent Co., Xi’an, China) were employed as monomer, cross-linker, catalyst, initiator, and dispersant, respectively. KOH, KCl, and ethanol (Xi’an Tianyi Chemical Reagent Co., China) were used to prepare the de-coring solution. Spherical aluminum powders (*D*_50_ = 40 μm, Hunan Ningxiang Jiweixin Metal Powder Co., Ltd., Changsha, China) were used as additives. SiO_2_ ceramic slurries with a high solid loading and low viscosity were obtained after using the Funk–Dinger (F–D) distribution function to design the volume fractions of SiO_2_ powders. The viscosities of ceramic slurries were well-maintained because of the high packing density of particles when the particle graduation number increased to four [25]. Five kinds of samples were formed with different amounts of Al (*D*_50_ = 40 µm) additives and the compositions are shown in Table 1. The Al additive replaced the content of SiO_2_ with a diameter of 40 µm in S2–S5 as they have similar size distributions. 

### 2.2. Gelcasting Process

**Figure 1:**
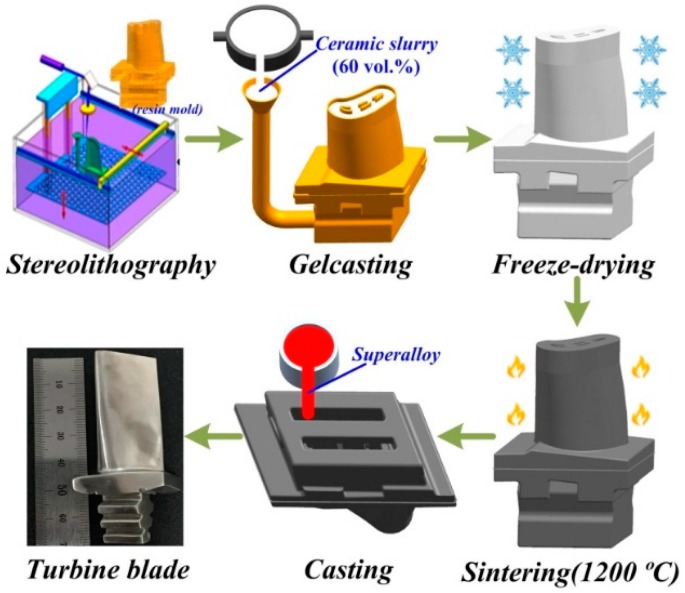
Fabrication process of SiO_2_-based ceramic molds.

The detailed specific manufacturing procedures of ceramic molds by SL and gelcasting methods are shown in Figure 1. SL prototypes for ceramic molds were prepared by an SL device (SPS600B, Xi’an Jiaotong University, Xi’an, China) using a photosensitive resin (SPR 8981, Zhengbang Technology Co., Ltd., Zhuhai, China). Ceramic molds and samples were made by aqueous gelcasting of SiO_2_ ceramic with different additives. The premix solution with 15% concentration was obtained by dissolving the AM and the MBAM in a 24:1 ratio. A 2 wt % sodium polyacrylate was added as a dispersant. After ball milling for 40 min, ceramic slurry with a solid loading of 60 vol % was obtained. After the initiator and catalyst were added, the ceramic slurry with a viscosity below 1 Pa·s was poured into the SL prototype and polymerized to form a green body. The green ceramic molds were vacuum freeze-dried for 24 h which could help to reduce shrinkage and avoid drying cracks [26]. High temperature could accelerate the mullitization conversion as the values of nucleation and growth for mullite crystallization [27] are connected to the incubation time (τ) and the rate constant (*k*), respectively. The activation energy for nucleation $E_{a}^{N}$ was calculated from Equation (1):

(1) $${\tau =\tau }_{0}\exp\left(E_{a}^{N}/RT\right)$$

The activation energy for nucleation and growth ($E_{a}^{NG}$) was calculated using Equation (2):

(2) $$k=k_{0}\exp\left(-E_{a}^{NG}/RT\right)$$

where τ_0_ is a constant, *R* the gas constant, T the absolute temperature, and *k*_0_ a constant. Obviously, with the increase of temperature (*T*), the incubation time (τ) decreased and rate constant (*k*) increased. It was deduced that the high temperature promoted the mullitization. Therefore, the green ceramic molds were sintered at 1200 °C and kept for 3 h to further synthesize mullite whiskers [26,28]. The frequently employed AM and MBAM offered the gelcast SiO_2_ ceramic molds a relative green strength of over 30.0 MPa to maintain the integrity of the ceramic molds. Operators were prevented from having direct contact with the toxic AM, and tail gas treatment reduced the pollution from AM when sintering [29,30,31,32]. Ceramic molds, bending strength test samples (10 mm × 4 mm × 60 mm), and creep deformation test samples (Φ50 mm × Φ12.5 mm × 50 mm) were obtained in the same way.

### 2.3. Measurements

The high-temperature strength was measured by a three-point bending test machine (HSST-6003QP, Sinosteel Luoyang Institute of Refractories, Luoyang, China), and each data point represented the average value of six individual tests at 1200 °C. Creep deformation was tested by a high-temperature Rul–Creep Tester (HRY-03P/Y, Sinosteel Luoyang Institute of Refractories, Luoyang, China) at 1200 °C. The porosity and density were measured by the Archimedes method. Microstructures and whiskers were observed by scanning electron microscopy (SEM) (SU-8010, Hitachi Ltd., Tokyo, Japan) and transmission electron microscopy (TEM) (JEM-2100Plus, JEOL in Tokyo, Japan), respectively. The phase compositions were identified by X-ray diffraction (XRD) (X’Pert Protype, Panalytical BV, Almelo, the Netherlands), and the composition of whiskers was analyzed by energy-dispersive spectroscopy (EDS). Thermogravimetric analysis and differential scanning calorimetry (TG-DSC) (STA449C, Netzsch Company, Selb, Germany) were used to inspect the sintering behaviors of ceramic molds. Distortions of internal structure were observed by X-ray scanning (Y. Cheetah, YXLON Ltd., Hamburg, Germany). Fracture toughness was tested by an electronic universal testing machine (SANS CMT4304, Xi’an, China).

## 3. Results and Discussion

### 3.1. Synthesis of Mullite Whiskers

#### 3.1.1. VLS Growth Mechanism

**Figure 2:**
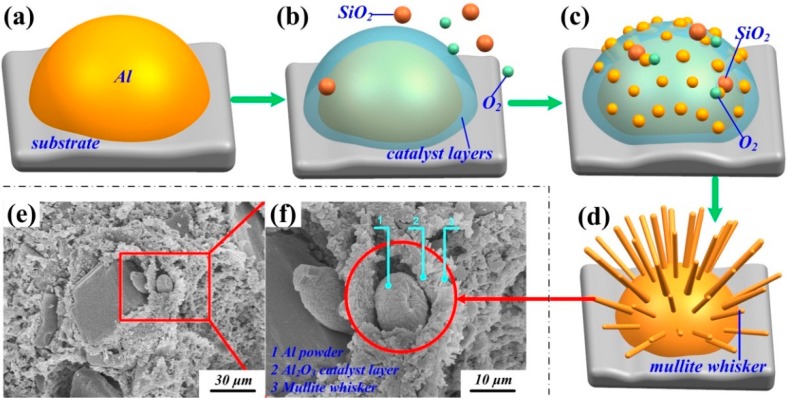
(**a**–**d**) Vapor–liquid–solid (VLS) growth process of mullite whiskers; (**e**) Scanning electron microscopy (SEM) morphology of SiO_2_ ceramic; (**f**) Microstructure of SiO_2_ ceramic.

Figure 2 illustrates the growth mechanism of urchin-like mullite whiskers using VLS growth method. Al powder additive was added to promote the synthesis of mullite whiskers. Al powder started to melt at 668 °C. With the increase of temperature, liquid Al began to evaporate, and then Al vapor diffused outward. An Al_2_O_3_ catalyst layer was formed on the surfaces through oxidation. Figure 2b shows that the Al_2_O_3_ catalyst layer worked as a catalytic and transition layer to promote the reaction among Al, SiO_2_, and O_2_. Figure 2c shows that the mullitization reaction gradually occurred above 998 °C. The high temperature provided enough energy for liquid Al to move from the substrate to the top of the whisker, promoting the growing of whiskers based on the Kirkendall principle [8]. After being sintered at 1200 °C, the urchin-like mullite whiskers were generated through the VLS growth method. Figure 2e shows the morphology of the sample after being sintered at 1100 °C. Al powder, Al_2_O_3_ catalyst layer, and mullite whiskers are found in Figure 2f, confirming the synthesis mechanism of mullite whiskers.

**Figure 3:**
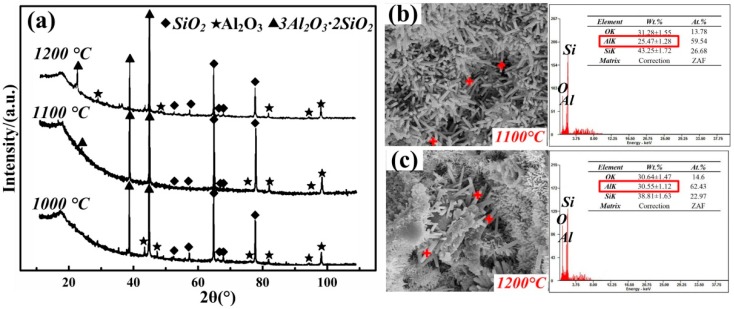
(**a**) X-ray diffraction (XRD) patterns of sample 4 at 1000, 1100, and 1200 °C. (**b**,**c**) Energy spectrum analysis of whiskers at 1100 and 1200 °C, respectively.

Figure 3a shows the XRD patterns of sample 4, which was sintered at 1000, 1100, and 1200 °C. Al_2_O_3_, SiO_2_, and mullite were observed in these patterns, and no other phase was detected. The varying intensity of mullite peaks in the XRD analysis shown in Figure 3a reveal that the mullitization conversion was higher at 1200 °C. Figure 3b,c show the morphology of whiskers. According to the energy spectrum analysis, the mass content of Al in the whiskers significantly increased from 25.47 to 30.55 wt % as the sintering temperature increased from 1100 to 1200 °C, showing an increased yield of mullite whiskers, which was consistent with the activation energy for nucleation and growth prediction mentioned above. The Al additive worked as a sintering assistant which promoted the synthesis of mullite whiskers in SiO_2_-based ceramic.

#### 3.1.2. Thermal Analysis

**Figure 4:**
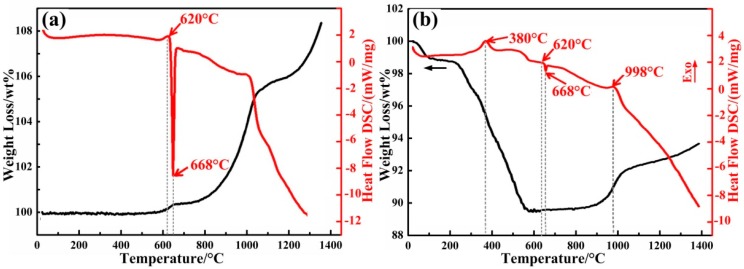
Differential scanning calorimetry (DSC) and thermal gravimetric analysis (TG) heating curves: (**a**) TG/DSC curves for the Al powder; (**b**) TG/DSC curves for the sample with a 15 vol % Al additive.

Figure 4a shows the TG-DSC results for Al powder in air. The exothermic peak at 620 °C was observed in the differential scanning calorimetry (DSC) curves, which represented the surface oxidation of the Al. An endothermic peak observed at 668 °C was attributed to the melting of Al powder. After that, the oxidation reaction rate increased, and the corresponding weight increase of the sample was observed in the thermal gravimetric analysis (TG) curve. Figure 4b shows the thermal behavior (TG/DSC) of the SiO_2_-based sample with a 15 vol % Al additive. The exothermic peak observed at 380 °C was due to the thermal decomposition of gel networks. Then, the melting of Al and the oxidation reaction occurred, which promoted the formation of the Al_2_O_3_ catalyst layer shown in Figure 2b. The exothermic peak observed at 998 °C was mainly attributed to the reaction among Al, SiO_2_, and O_2_. After that, the mullite whiskers were gradually formed with an increase in mass of the sample. The detailed synthesis reaction of mullite whiskers is described as follows:

(3) $${2\mathrm{Al}+3O}_{2}\to 2{\mathrm{Al}}_{2}O_{3}$$

(4) $${3\mathrm{Al}}_{2}O_{3}{+2\mathrm{SiO}}_{2}\to 3{\mathrm{Al}}_{2}O_{3}\cdot {2\mathrm{SiO}}_{2}$$

In this study, the sintering temperature was set as 1200 °C (above 998 °C), which guaranteed that the raw materials would react completely.

### 3.2. Microstructure Analysis

**Figure 5:**
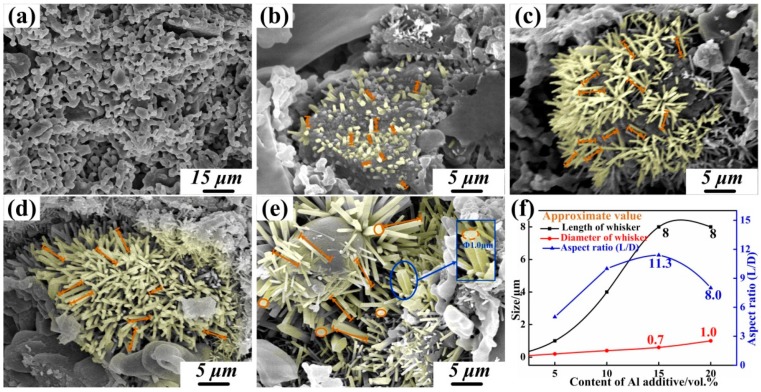
(**a**–**e**) Scanning electron microscopy (SEM) morphology of mullite whiskers with 0, 5, 10, 15, and 20 vol % Al additive, respectively; (**f**) Size distribution of whiskers.

Figure 5 shows the morphology of the samples with 0, 5, 10, 15, and 20 vol % Al additives. After sintering at 1200 °C, urchin-like mullite whiskers grew in gaps and formed bridge structures between particles. Figure 5a shows that there were no mullite whiskers observed on the fracture surface in sample 1. As shown in Figure 5b, when 5 vol % Al was added, the length of whiskers was less than 1.0 μm and the diameter of the whiskers was nearly 0.2 μm. As shown in Figure 5c, whiskers with a length in the range of 3~6 μm and a diameter of 0.4 μm were synthesized in the ceramic when the additive of Al was 10.0 vol %. Figure 5d shows that urchin-like mullite whiskers with the length in the range of 5~8 μm and a diameter of 0.7 μm bridged the walls when 15 vol % Al was added. Figure 5b–f proves that the formed whiskers grew longer and thicker with an increasing additive amount, and the aspect ratio of length and diameter of the urchin-like mullite whiskers also increased. The increasing amount and enlarged size of the whiskers contributed to the reinforcement of the SiO_2_ ceramic, since larger whiskers play a more remarkable role as bridges between particles. However, when the Al additive was further increased to 20 vol %, the length of whiskers no longer increased, which limited the bridge effect of the whiskers, while the diameter of the whiskers continued to increase to 1.0 μm (Figure 5e). Meanwhile, the aspect ratio of length and diameter of the urchin-like mullite whiskers decreased (Figure 5f).

**Figure 6:**
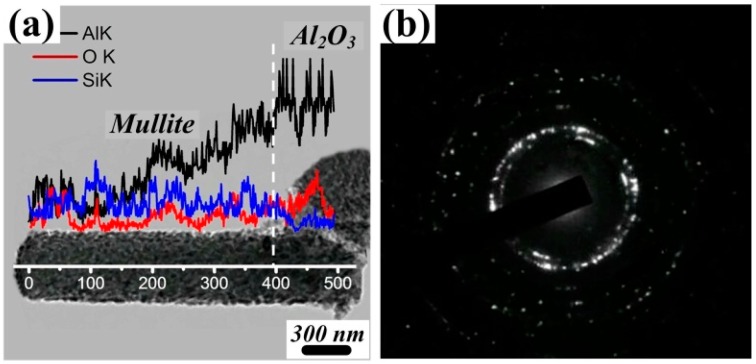
(**a**) Transmission electron microscopy (TEM) morphology of mullite whiskers; (**b**) Diffraction pattern of whiskers.

Figure 6 shows the TEM photograph of mullite whiskers from the VLS growth method. Figure 6a shows the bright field image of whiskers. Combined with EDS elemental analysis, the trunk of the whisker was found to be mainly composed of Al, Si, and O elements, which were consistent with that of mullite. Meanwhile, the bottom of the whisker (containing Al and O elements) was connected to the Al_2_O_3_ catalyst layer where the whiskers were driven to grow upward. Spot patterns in Figure 6b reveal that polycrystalline mullite whiskers were formed in the SiO_2_-based ceramic (Figure 6b).

### 3.3. High-Temperature Performance Analysis

**Figure 7:**
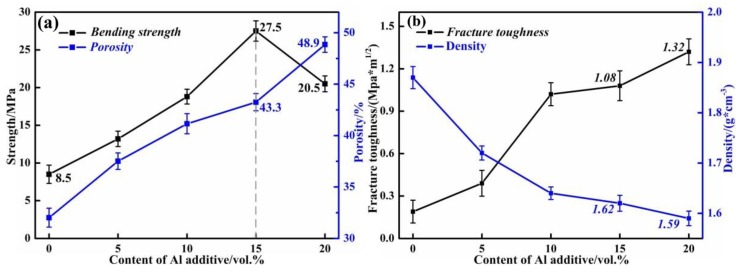
(**a**) High-temperature strength and porosity distribution of SiO_2_ ceramic molds; (**b**) Fracture toughness and density distribution of SiO_2_ ceramic molds.

The mechanical properties are closely related to the microstructures and phases. Figure 7a shows the high-temperature strength at 1200 °C and open porosities of the ceramic molds. The high-temperature strength of sample 1 was only 8.5 MPa when there was no Al additive. The increasing amount of Al additive helped to improve the high-temperature strength of SiO_2_-based ceramic molds. Then, a maximum high-temperature strength of 27.5 MPa was obtained in sample 4 when 15 vol % Al was added. This result was attributed to the generation of mullite (as confirmed by XRD analysis) and the bridging effect of mullite whiskers. The high-temperature strength of SiO_2_-based ceramic mold could be guaranteed in two ways: (1) Via bridge effects of mullite whiskers between particles and (2) by the reduction in porosity. However, the open porosity also increased with the increase of the Al additive. When the content of Al additive increased from 15 to 20 vol %, the porosity of the ceramic mold increased from 43.3% to 48.9%. In this process, the high-temperature strength decreased from 27.5 to 20.5 MPa because the increased bridging effect could not compensate for the loss of strength caused by high porosity. Figure 7b shows the fracture toughness and density distribution of SiO_2_ ceramic molds after being sintered at 1200 °C. Obviously, the fracture toughness of SiO_2_ ceramic molds increased with an increasing additive amount, and a fracture toughness of over 1.0 MPa.m^1/2^ was obtained when 15 vol % Al was added. The synthesized mullite whiskers and bridging effect promoted the improvements of fracture toughness. Meanwhile, the density of SiO_2_ ceramic molds decreased with an increasing porosity of the ceramic molds. In general, the density was maintained around 1.60 g·cm^−3^ when the Al additive was over 10 vol %.

**Figure 8:**
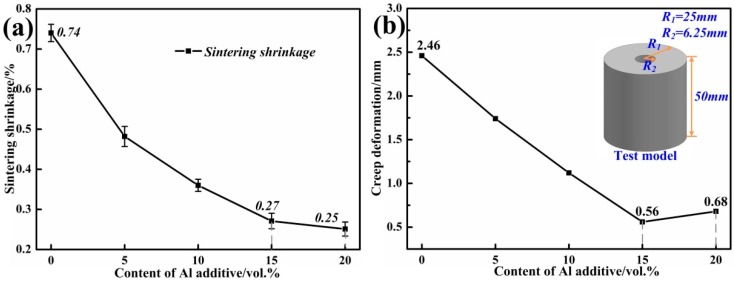
(**a**) Sintering shrinkage of SiO_2_ ceramic molds with 0, 5, 10, 15, and 20 vol % Al additive; (**b**) Creep deformation of SiO_2_ ceramic molds with 0, 5, 10, 15, and 20 vol % Al additive.

Figure 8a shows the sintering shrinkage of samples 1–5 at 1200 °C. Al powder, exhibiting a 28% volume expansion during the oxidation reaction (Al→α-Al_2_O_3_), was applied to an accelerating sintering reaction to further compensate for the shrinkage upon sintering [23]. Obviously, the sintering shrinkage decreased with the increase of Al additive. When 15 or 20 vol % Al was added, the sintering shrinkage was below 0.3%. Low shrinkage could guarantee the precision of ceramic mold and metal casting. Figure 8b shows the creep deformation of samples 1–5 at 1200 °C. Creep deformation reduced with the increase of Al additive which promoted the sintering reaction of ceramic particles. The minimum creep deformation was only 0.56 mm in sample 4 when 15 vol % Al was added. As mentioned above, when 20 vol % Al was added, the length of whiskers no longer increased whilst the porosity of the ceramic continued to rise, so the creep deformation increased to 0.68 mm in sample 5.

**Figure 9:**
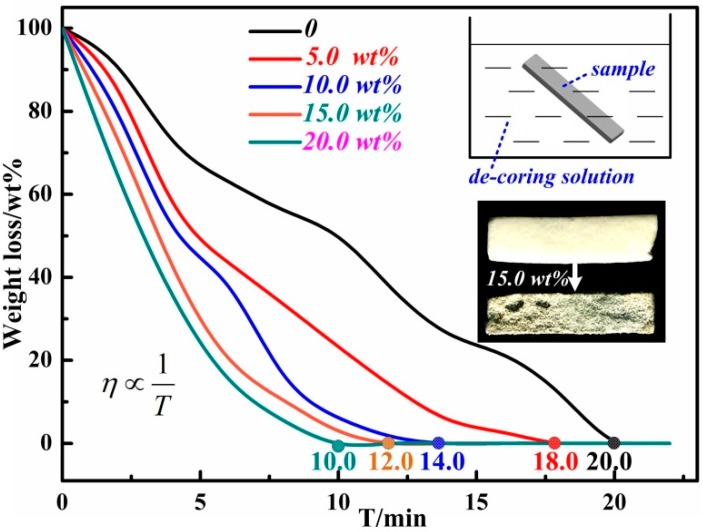
De-coring rate of SiO_2_ ceramic molds with 0, 5, 10, 15, and 20 vol % Al additive.

Good leachability is a key advantage of SiO_2_-based ceramic molds in the manufacture of hollow turbine blades. The as-shaped ceramic core samples were immerged into the etchant KOH solution. Figure 9 shows the de-coring rate of SiO_2_-based ceramic cores. Previous research proved that a 70 wt % KOH solution supplemented with 10 wt % KCl and ethanol obtained a high de-coring rate in the removal process and had little corrosion to the metal blade [33]. The basic parameter of the removal process of a SiO_2_-based ceramic core was studied at room temperature and normal pressure. The de-coring rate (*v*) is calculated by Equation (5) by measuring the dry weights before and after the removal process [33].

(5) $$v=\frac{m_{1}-m_{2}}{t\times m_{1}}\times 100\%$$

where *v* has the unit wt %·h^−1^, *m*_1_ and *m*_2_ are the weights of the sample before and after the removal process, respectively, and t is the etching time. Combined with the data in Figure 9, the final de-coring rates of S1–S5 were obtained to be 3.0, 3.3, 4.3, 5.0, and 6.0 wt %·h^−1^, respectively. In this study, the synthesized mullite whiskers and high porosity accelerated the de-coring rate of ceramic samples. Therefore, the de-coring rates of S4 and S5 were accelerated by 67% and 100%, respectively, compared to that of S1 representing the pure SiO_2_-based ceramic. The high content Al additive can accelerate the alkali removal process of a SiO_2_-based ceramic core and promote the rapid manufacturing of blades.

### 3.4. Case Study

**Figure 10:**
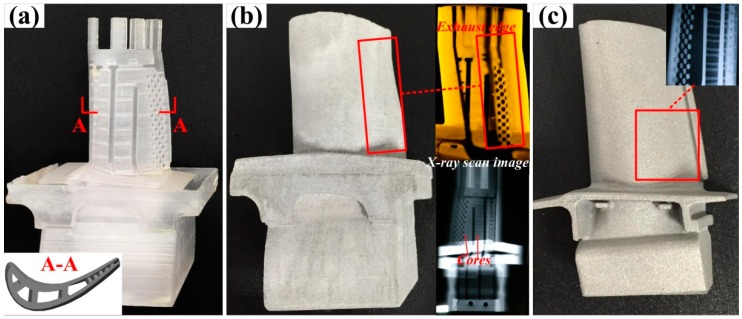
Fabrication of a hollow turbine blade: (**a**) Resin mold; (**b**) Mullite whisker-reinforced ceramic mold with X-ray inspection; (**c**) Hollow turbine blade.

As shown in Figure 10, a SiO_2_-based ceramic mold (Figure 10b) with 15 vol % Al additive was successfully fabricated by gelcasting in combination with a stereolithography resin mold (Figure 10a). The tiny internal structure of the ceramic mold was well-maintained. A hollow turbine blade (Figure 10c) was cast using the SiO_2_-based ceramic mold, indicating that urchin-like mullite whiskers synthesized through the VLS growth method were effective in improving the high-temperature performance of porous SiO_2_-based ceramic molds which meet the requirements of investment casting for hollow turbine blades.

## 4. Conclusions

In this study, the high-temperature performance of SiO_2_-based ceramic molds was significantly improved by the use of urchin-like mullite whiskers synthesized using the VLS growth method. The SiO_2_-based ceramic molds could meet the requirements of precision casting for hollow turbine blades. The major findings of this study are as follows:

(1) Polycrystalline urchin-like mullite whiskers were successfully synthesized through the VLS growth method. Urchin-like mullite whiskers were significantly effective in improving the high-temperature strength of SiO_2_-based ceramic molds through the bridge effect. The SiO_2_-based ceramic mold obtained a maximum bending strength of 27.5 MPa with a 15 vol % Al additive.
(2) When 15 vol% Al was added to the raw material, a minimum creep deformation of 0.56 mm and a sintering shrinkage below 0.3% were obtained, and the de-coring rate increased by 67%. An integral ceramic mold for a hollow turbine blade was successfully fabricated.

In conclusion, urchin-like mullite whiskers could be used to enhance the high-temperature performance of SiO_2_-based ceramic molds and enhance the fabrication of complex hollow turbine blades. This method has great potential applications in the precise fabrication of ceramic and the field of metal casting. 

## Figures and Tables

**Table 1:** Compositions of gelcasting SiO_2_-based samples.

Sample	No. Weight Fraction (vol %)				
	SiO_2_ (100 µm)	SiO_2_ (40 µm)	SiO_2_ (5 µm)	SiO_2_ (2 µm)	Al (40 µm)
S1	22.1	40.9	21.6	14.4	0
S2	22.1	35.9	21.6	14.4	5.0
S3	22.1	30.9	21.6	14.4	10.0
S4	22.1	25.9	21.6	14.4	15.0
S5	22.1	20.9	21.6	14.4	20.0
